# Fast Simultaneous Determination of 13 Nucleosides and Nucleobases in *Cordyceps sinensis* by UHPLC–ESI–MS/MS

**DOI:** 10.3390/molecules201219807

**Published:** 2015-12-04

**Authors:** Shi-Yu Zong, Han Han, Bing Wang, Ning Li, Tina Ting-Xia Dong, Tong Zhang, Karl W. K. Tsim

**Affiliations:** 1 School of Pharmacy, Shanghai University of Traditional Chinese Medicine, No. 1200 Cailun Road, Pudong New District, Shanghai 201203, China; zongsy114@163.com; 2 Experimental Center of Teaching and Learning, Shanghai University of Traditional Chinese Medicine, No. 1200 Cailun Road, Pudong New District, Shanghai 201203, China; pashanhan@163.com; 3 Division of Life Science and Center for Chinese Medicine, The Hong Kong University of Science and Technology, Clear Water Bay Road, Hong Kong, China; mailtolining@gmail.com (N.L.); botina@ust.hk (T.T.-X.D.); botsim@ust.hk (K.W.K.T.)

**Keywords:** *Cordyceps sinensis*, nucleosides, nucleobases, UHPLC–ESI–MS/MS

## Abstract

A reliable ultra-high-performance liquid chromatography–electrospray ionization–tandem mass spectrometry (UHPLC–ESI–MS/MS) method for the fast simultaneous determination of 13 nucleosides and nucleobases in *Cordyceps sinensis* (*C. sinensis*) with 2-chloroadenosine as internal standard was developed and validated. Samples were ultrasonically extracted in an ice bath thrice, and the optimum analyte separation was performed on an ACQUITY UPLC^TM^ HSS C18 column (100 mm × 2.1 mm, 1.8 μm) with gradient elution. All targeted analytes were separated in 5.5 min. Furthermore, all calibration curves showed good linear regression (*r* > 0.9970) within the test ranges, and the limits of quantitation and detection of the 13 analytes were less than 150 and 75 ng/mL, respectively. The relative standard deviations (RSDs) of intra- and inter-day precisions were <6.23%. Recoveries of the quantified analytes ranged within 85.3%–117.3%, with RSD < 6.18%. The developed UHPLC–ESI–MS/MS method was successfully applied to determine nucleosides and nucleobases in 11 batches of *C. sinensis* samples from different regions in China. The range for the total content in the analyzed samples was 1329–2057 µg/g.

## 1. Introduction

*Cordyceps*, commonly called “*Dong Chong Xia Cao*” (winter worm summer grass) [1], is a complex of the stromata of the ergot fungus, *Cordyceps sinensis* (*C. sinensis*) (Berk.) Sacc. parasitized on the larva of *hepialidae* insects, and dead caterpillar [2]. *C. sinensis* is an entomogenous fungus that belongs to class *Pyrenomycetes*, order *Clavicipitales*, family *Clavicipitaceae* [3]. In 1694, *Cordyceps* was first recorded by Wang Ang in “Ben Cao Bei Yao” and described as “*Cordyceps sinensis*, Gan Ping, protecting lung and benefiting kidney, hemostatic phlegm, cough has pain”. “*Cordyceps* derived from Jiading of Sichuan, shows the highest quality” [4]. *Cordyceps* can nourish the lung and kidney, alleviate the symptoms of kidney deficiency, and treat hemostatic phlegm, impotence, spermatorrhea, waist and knee pain, cough asthenia, cough and hemoptysis [5]. *C. sinensis* have been used as a medicine for more than 300 years in China [4]. Studies have confirmed that *C. sinensis* has significant hypolipidemic, hypoglycemic, immunoregulation, antioxidation, antitumor, antifibrosis, antiarrhythmia, anti-inflammatory and other effects [2,6,7,8,9]. *C. sinensis* is mainly distributed in Qinghai, Gansu, Sichuan, Tibet, Yunnan and other high-altitude (3500–5000 m) grassland soil in China [1,10,11]. *C. sinensis* is a very expensive and valuable Chinese herbal medicine whose market price is as high as hundreds of thousands of RMB a kilo. It is also an edible herb that is widely used as a tonic medicine, and the market demand is very strong.

*C. sinensis* contains many chemical constituents. Nucleosides are recognized as the main bioactive components [4,8] and are used as chemical markers for quality control [12]. Nucleosides have antiplatelet-aggregation, antioxidative, antiarrhythmia, anticonvulsion [13,14,15], and antitumor [16] activities. Nucleosides also play an important role in the human body’s metabolism, immunity, cardiovascular system, liver, kidney, and nervous system [17,18,19,20]. Therefore, establishing a rapid, sensitive, and reliable nucleoside-analysis method is very important for the full development and utilization of the rare resource *C. sinensis*.

Currently available analysis methods for nucleosides and nucleobases in *Cordyceps* mainly include high-performance liquid chromatography (HPLC)–ultraviolet spectroscopy [21], matrix solid-phase dispersion–HPLC [22], capillary electrophoresis [23,24], and ultrahigh-performance liquid chromatography (UHPLC)–photodiode array [25]. However, these methods suffer from the limited sensitivity of UV detectors. In recent years, HPLC combined with mass spectrometry (MS) has been widely used to determine nucleosides and nucleobases, such as liquid chromatography (LC)–MS [26,27], ion-pairing reversed-phase LC–MS [28,29], and hydrophilic interaction chromatography (HILIC)–electrospray ionization (ESI)–MS [10]. Nevertheless, few components can be determined, the analysis time is long, and the sample-preparation process often involves a high-temperature extraction step, which greatly influences the recovery of nucleosides due to the thermal instability [30,31].

In the present study, ultrasonic multiple extraction in an ice bath was performed to effectively avoid the content change of target components caused by increased temperature without affecting the extraction rate. The subsequent UHPLC–ESI–tandem spectroscopy (MS/MS) method improved the sensitivity and greatly shortened the analysis time, thereby enabling the quantitative analysis of 13 analytes in 5.5 min.

## 2. Results and Discussion

### 2.1. Optimization of UHPLC Conditions

**Figure 1:**
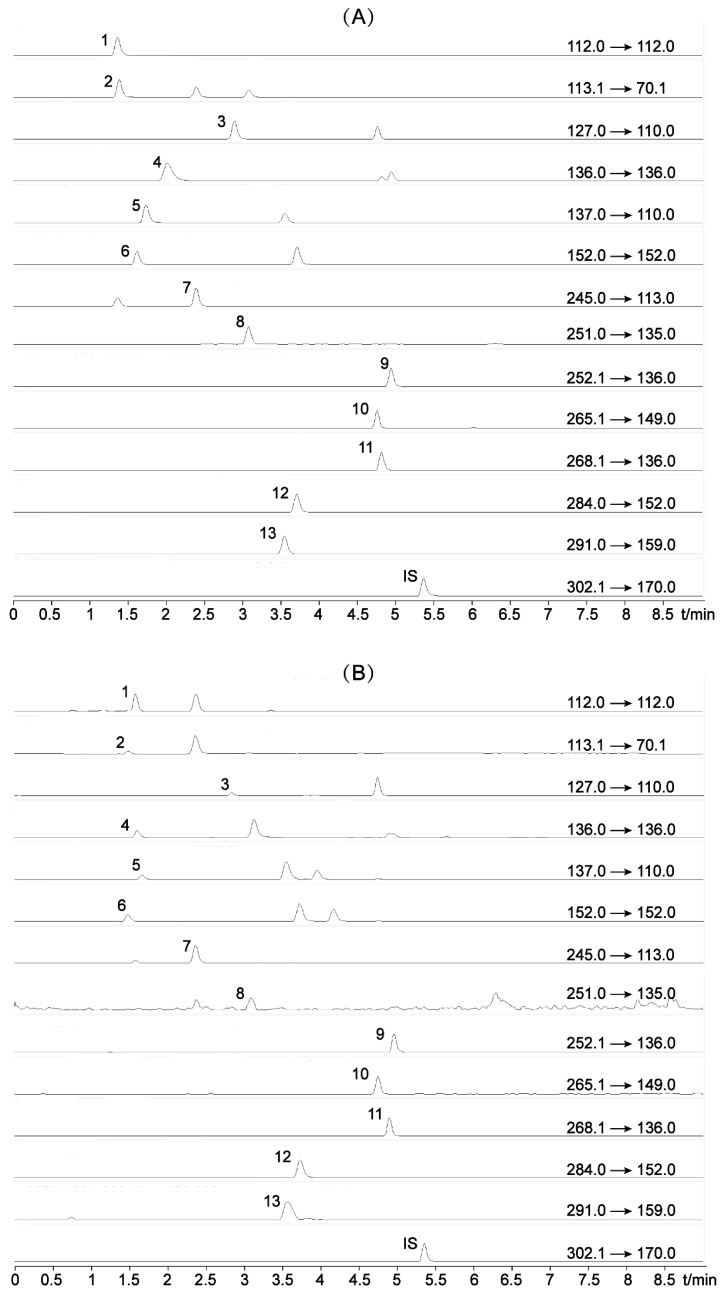
Typical ultrahigh-performance liquid chromatograms (UHPLC) of (**A**) mixed standards and (**B**) representative sample with multiple reaction monitoring (MRM) method (1) Cytosine; (2) Uracil; (3) Thymine; (4) Adenine; (5) Hypoxanthine; (6) Guanine; (7) Uridine; (8) 2′-Deoxyuridine; (9) Cordycepin; (10) Thymidine; (11) Adenosine; (12) Guanosine; (13) Inosine; and 2-chloroadenosine (internal standard, IS).

Given their similar chemical structures, nucleoside compounds are difficult to separate from one another. Thus, in this work, we selected water–acetonitrile and 0.1% formic acid–acetonitrile as the mobile phase for gradient elution. Results showed that water–acetonitrile led to better separation and peak shapes than 0.1% formic acid–acetonitrile. In addition, the separation abilities of three columns (*i.e*., a Waters ACQUITY UPLC^TM^ HSS C18 column (100 mm × 2.1 mm, 1.8 μm), a TSK-GEL ODS-140HTP column (100 mm × 2.1 mm, 2.3 μm), and an Aglient ZORBAX SB-Aq column (50 mm × 4.6 mm, 5 μm)) were compared. Results indicated that the first one had better retention and separation abilities for these hydrophilic analytes than the other two when using the same mobile phase. Moreover, the entire analysis process took 9 min, and all target analytes were separated in 5.5 min, which was significantly shorter than any previously reported LC–MS method. The typical chromatograms of the 13 analytes in multiple reaction monitoring (MRM) mode are presented in Figure 1.

### 2.2. Optimization of ESI–MS/MS Conditions

The MS conditions of 13 target components were optimized, and results showed that the positive-ion mode was more sensitive. Regarding the monitoring mode, the abundance of product ions for cytosine, adenine, and guanine were too low for MRM detection. Thus, selective-ion monitoring (SIM) was used for the quantitative determination of these three nucleobases. The other 10 target components were analyzed in MRM mode. In addition, other MS parameters such as fragmentation amplitude, cone voltage, and collision energy were also subjected to adjustment and optimization to achieve the maximum sensitivity. The optimum ESI–MS/MS parameters are shown in Table 1.

**Table 1:** Electrospray ionization-tandem spectroscopy (ESI-MS/MS) ions (positive ionization mode) of 13 analytes in *Cordyceps sinensis*.

Analyte	Precursor Ion (*m*/*z*)	Product Ion (*m*/*z*)	Fragmentor	CE	CVA	Monitoring Mode
Cytosine	112.0	-	180	0	0	SIM
Uracil	113.1	70.1	100	16	0	MRM
Thymine	127.0	110.0	100	16	0	MRM
Adenine	136.0	-	120	0	0	SIM
Hypoxanthine	137.0	110.0	135	20	5	MRM
Guanine	152.0	-	120	0	0	SIM
Uridine	245.0	113.0	85	2	5	MRM
2′-Deoxyuridine	251.0	135.0	90	7	5	MRM
Cordycepin	252.1	136.0	80	15	4	MRM
Thymidine	265.1	149.0	100	10	4	MRM
Adenosine	268.1	136.0	100	16	3	MRM
Guanosine	284.0	152.0	70	7	5	MRM
Inosine	291.0	159.0	90	10	1	MRM

### 2.3. Optimization of Sample Preparation

Two extraction methods, heat reflux (85 °C) and ice bath ultrasonic method were compared. Results showed that the contents of nucleosides and their bases, extracted with heat reflux were significantly higher than those yielded with ultrasonic extraction in an ice bath. For instance, the contents of guanosine and uridine were 287 μg/g and 270 μg/g, after extraction by ice bath ultrasonic method, while 856 μg/g and 360 μg/g, were obtained by heat reflux, respectively. This was consistent with the results of a previous study [30]. The high temperature of heat reflux may degrade nucleic acids to nucleosides and bases [28]. Therefore, in order to reduce thermal effects on the content of nucleoside compounds, ultrasonic extraction in an ice bath was performed. Given that nucleosides and nucleobases are strongly polar compounds, choosing 10% aqueous methanol as extraction solvent could better extract the target components. The extraction time was also optimized to achieve the best extraction effect. First, in the case of the cordycepin-extraction rate, 1 h was allotted for each of three extraction times, and the extraction rates of each time were 4.77%, 46.63%, and 48.60%, respectively, which indicated that extraction was incomplete. Therefore, extraction time was prolonged to 2 h for each of three successive extraction cycles, and the extraction rates of each time were 64.13%, 29.53%, and 6.34%, indicating that the selected extraction method could ensure an almost complete extraction.

### 2.4. Calibration Curves, Limit of Detection (LOD), and Limit of Quantification (LOQ)

The calibration curve for each analyte was obtained in triplicate with at least seven appropriate concentrations. The regression equations for the 13 nucleosides and nucleobases were calculated in the form of *y* = A*x* + B, where *y* and *x* were the peak area ratios of analytes to IS and the corresponding analyte concentration injected, respectively. As shown in the results in Table 2, the correlation coefficients of all target components exceeded 0.9970 with good linearity. The LOD and LOQ of 13 analytes were measured at signal-to-noise ratios of 3 and 10, and the ranges were 0.04–75 and 0.1–150 ng/mL, respectively. This finding indicated that the method established in this study for the components cordycepin, 2′-deoxyuridine, adenosine, guanosine, and inosine detection, was more sensitive than previously reported HPLC–ESI–MS/MS [7] and HILIC–ESI–MS [10] methods. For instance, the LOD and LOQ values of adenosine were 0.20 and 0.50 ng/mL in this study, while 1.72 and 3.44 ng/mL and 0.60 and 2 ng/mL, respectively, were reported in the two studies mentioned above.

**Table 2:** Regression data, Limit of Detection (LOD), and Limit of Quantification (LOQ) for the 13 analytes.

Analyte	Regression Equation	*r*	Linear Range (µg/mL)	LOD (ng/mL)	LOQ (ng/mL)
Cytosine	*y* = 0.683*x* *−* 0.007	0.9995	0.010–2.486	1.00	10.00
Uracil	*y* = 0.048*x* *+* 0.022	0.9970	0.077–9.885	75.00	150.00
Thymine	*y* = 0.094*x* *+* 0.001	0.9995	0.038–4.904	20.00	41.00
Adenine	*y* = 2.611*x* *+* 0.001	0.9995	0.005–1.228	0.35	1.00
Hypoxanthine	*y* = 0.171*x* *+* 0.017	0.9990	0.019–4.875	0.40	1.00
Guanine	*y* = 2.297*x* *+* 0.008	0.9985	0.008–1.977	4.00	8.00
Uridine	*y* = 0.099*x* *−* 0.006	0.9980	0.076–9.750	10.00	19.00
2′-Deoxyuridine	*y* = 0.053*x* *+* 0.001	0.9995	0.039–4.952	2.00	8.00
Cordycepin	*y* = 1.845*x* *+* 0.062	0.9975	0.005–1.212	0.04	0.10
Thymidine	*y* = 0.052*x* *+* 0.002	0.9995	0.078–4.981	20.00	79.00
Adenosine	*y* = 1.624*x* *+* 0.021	0.9995	0.010–4.904	0.20	0.50
Guanosine	*y* = 0.766*x* *+* 0.061	0.9995	0.038–9.769	0.40	1.00
Inosine	*y* = 0.095*x* *+* 0.006	0.9980	0.0198–2.476	0.50	2.50

### 2.5. Method Validation

Intra- and inter-day variations were used to evaluate method precision. For the intra-day variabilitiy test, the mixed standard solutions were analyzed for six replicates within a day; for the inter-day variability test, the solutions were examined for three consecutive days. The relative standard deviation (RSD) was taken as a measure of precision, and results are listed in Table 3. Thirteen nucleosides and nucleobases had intra- and inter-day precision RSDs (*n* = 6) of less than 2.53% and 6.23%, respectively. The RSDs were also taken as measures of repeatability and stability. A stability test was further performed to analyze the variations in the sample solutions at 0, 2, 4, 8, 12, and 24 h. The overall stability and repeatability variations were less than 6.65% and 6.39%, respectively. A recovery test was also performed to validate the accuracy of the developed method by adding a known amount of the reference marker compounds into accurately weighed samples. The spiked samples were extracted according to Section 3.2 (Sample-preparation method), and then the developed UHPLC–ESI–MS/MS method was used for analysis. The results listed in Table 3 revealed that all analyte recoveries ranged within 85.3%–117.3%, with RSDs less than 6.18%. Thus, the developed method was accurate and reliable.

**Table 3:** Precision, repeatability, stability and recovery of the 13 analytes.

Analyte	Precision (RSD, %, *n* = 6)		Stability (RSD, %, *n* = 6)	Repeatability (RSD, %, *n* = 6)	Recovery (%, *n* = 3)	
	Intraday	Interday			Mean	RSD (%)
Cytosine	0.86	6.23	4.78	4.81	102.3	4.79
Uracil	1.21	5.73	3.11	5.92	117.3	2.46
Thymine	0.81	3.85	2.97	6.05	97.0	2.01
Adenine	0.70	4.73	2.87	5.88	109.7	3.08
Hypoxanthine	0.95	5.23	6.65	3.68	110.6	4.77
Guanine	1.27	3.61	2.38	5.82	85.7	2.42
Uridine	2.53	2.79	1.76	4.75	90.3	6.18
2′-Deoxyuridine	2.49	5.72	5.40	6.39	98.6	4.10
Cordycepin	0.65	2.22	2.04	5.49	115.2	1.30
Thymidine	1.74	5.09	4.74	5.80	87.0	1.77
Adenosine	0.78	0.08	3.00	4.45	108.7	3.39
Guanosine	1.18	4.61	0.71	5.64	85.3	5.96
Inosine	1.51	4.86	3.86	5.11	116.7	3.21

### 2.6. Application and Sample Analysis

The developed UHPLC–ESI–MS/MS method was subsequently applied to the simultaneous determination of 13 nucleosides and nucleobases in 11 batches of *C. sinensis* from different regions in China, and the results are summarized in Table 4. All target compounds including 13 nucleosides and nucleobases were detected in almost all samples, and the total content (1329–2057 µg/g) was high. However, large differences existed in the nucleoside-compound contents of *C. sinensis* with different origins. For instance, inosine in sample 8 was 4.24 times the amount in sample 11, and cordycepin in sample 11 was 9.6 times the amount in sample 1. This finding suggested that the growth environment, picking season, and storage method likely influenced the content of nucleoside compounds. As a result, quality differences existed among varying *C. sinensis* resources. If the content of total nucleosides needs to be considered, sample 3 (Yushu, Qinghai Province) would be the best choice. On the other hand, if the focus is on a specific nucleoside component, such as adenosine, sample 11 (Naqu, Tibet) would be more suitable. In addition, the contents of guanosine, uridine, and adenosine were comparatively high in the samples, and the result was consistent with previous data [7,10,32,33]. Among them, adenosine has been used for the quality control index of *C. sinensis* [34], the contents of two other active ingredients guanosine and uridine were high in most of the samples. Thus, guanosine and uridine could also be considered as quality indices, to provide a wide range of options for the quality assessment of *C. sinensis*.

**Table 4:** Contents ^a^ (μg/g) of 13 nucleosides and nucleobases in 11 batches of *C. sinensis* samples (*n* = 3).

Analyte	1	2	3	4	5	6	7	8	9	10	11
Cytosine	85.2	110	39.5	60.4	48.8	57.4	49.1	26.4	90.6	299	155
Uracil	tr	nd	nd	nd	nd	nd	nd	11.3	nd	nd	tr
Thymine	12.5	12.0	11.8	11.6	11.6	11.4	12.0	nd	11.8	11.8	4.92
Adenine	66.0	60.2	69.1	74.5	46.8	68.5	53.9	18.9	42.0	43.2	4.43
Hypoxanthine	96.0	92.9	35.7	30.6	61.2	22.7	44.8	83.9	54.3	69.4	57.7
Guanine	1.14	tr	0.35	0.09	0.79	tr	0.40	nd	1.96	5.25	3.63
Uridine	618	495	730	626	495	490	627	403	625	415	270
2′-Deoxyuridine	2.61	2.97	6.59	5.92	3.43	3.08	5.96	2.76	1.35	tr	tr
Cordycepin	10.6	32.1	33.2	34.1	30.1	24.3	35.8	15.6	20.6	44.2	102
Thymidine	3.07	4.37	3.61	6.90	2.96	3.63	1.57	2.86	2.17	0.85	4.75
Adenosine	99.6	200	265	164	180	172	241	160	158	216	381
Guanosine	657	613	721	644	582	484	832	420	589	585	287
Inosine	115	92.7	142	107	145	151	123	250	201	154	59.1
Total	1767	1716	2057	1765	1608	1488	2026	1395	1797	1843	1329

^a^ The data are presented as the average of three replicates (RSDs < 7%); nd, not detected; tr, under the limits of quantitation.

## 3. Experimental Section

### 3.1. Materials and Standards

**Figure 2:**
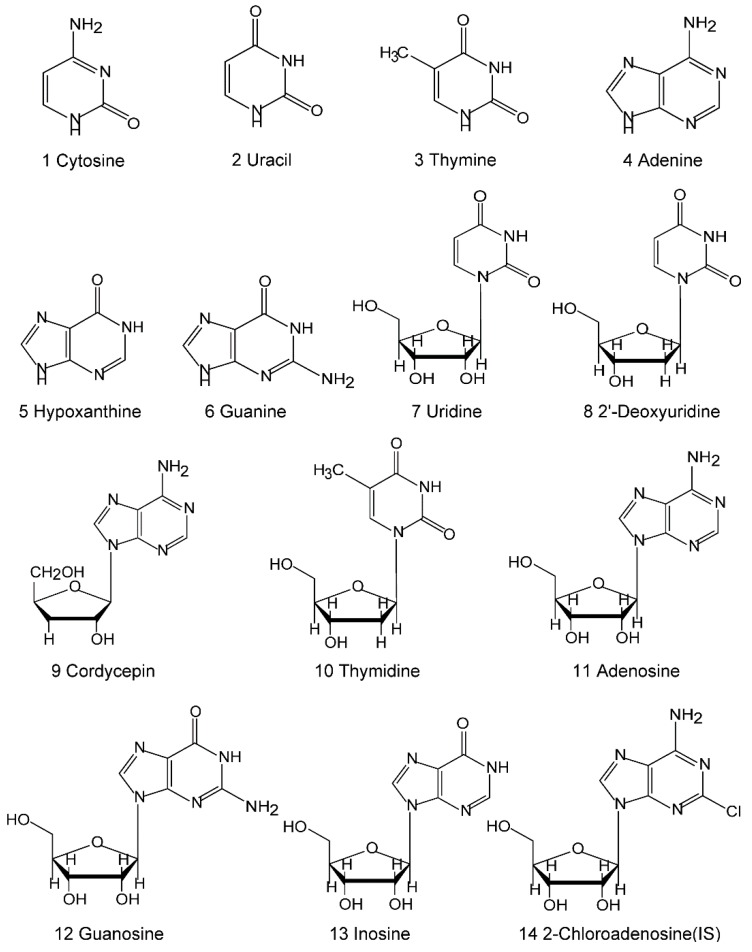
Chemical structures of the nucleosides and nucleobases identified in this study.

Acetonitrile and methanol were HPLC grade and obtained from Merck (Darmstadt, Germany). Deionized water was purified by Milli-Q system (Millipore, Bedford, MA, USA). Standards of cytosine, uracil, thymine, adenine, hypoxanthine, guanine, uridine, 2′-deoxyuridine, cordycepin, thymidine, adenosine, guanosine, and inosine were purchased from Sigma (St. Louis, MO, USA). The purity of each compound was not less than 98%, as determined by HPLC analysis. Their chemical structures are shown in Figure 2.

Eleven batches of *C. sinensis* samples were collected from different regions in China and numbered 1 to 11. Detailed origin information is listed in Table 5. All samples were authenticated by Prof. Ting Xia Dong (The Hong Kong University of Science and Technology, Hong Kong, China) as the *Clavicipitaceae* fungus *C. sinensis* (Berk.) Sacc. The voucher specimens were deposited at the Traditional Chinese Medicine Research and Development Center, The Hong Kong University of Science and Technology, Hong Kong, China.

**Table 5:** Regions of the 11 batches of *C. sinensis* samples.

Sample	Region	Sample	Region
1	Yushu, Qinghai Province	7	Naqu, Tibet
2	Yushu, Qinghai Province	8	Gannan, Gansu Province
3	Yushu, Qinghai Province	9	Linzhi, Tibet
4	Jiazha, Tibet	10	Changdu, Tibet
5	Naqu, Tibet	11	Naqu, Tibet
6	Ganzi, Sichuan Province	-	-

### 3.2. Sample Preparation

About 10 mL of 10% (*v*/*v*) aqueous methanol was added to 0.2 g of *C. sinensis*, and the mixture was evenly ground at 13,000 rpm by using a high-shear-dispersion emulsifying machine (Fluko Equipment Shanghai Co., Ltd., Shanghai, China). The mixture was then transferred into a glass tube with a stopper and ultrasonically extracted in an ice-bath (Kunshan Ultrasonic Instrument Co., Ltd., Kunshan, China, 40 kHz) 2 h for three consecutive times. After centrifugation (12,000 rpm; 20 min; room temperature), the supernatant was filtered, and analyte concentration was adjusted between the linearity ranges based on the preliminary determination. 2-Chloroadenosine (1 µg/mL) was used as internal standard for the quantitative determination of analytes by using a calibration curve. All solutions were stored at 4 °C and passed through a 0.22 μm membrane prior to injection.

### 3.3. UHPLC Conditions

For the UHPLC analysis, an ACQUITY UPLC^TM^ HSS C18 column (100 mm × 2.1 mm, 1.8 μm, Waters Co., Milford, MA, USA) was used. The mobile-phase components acetonitrile (A) and water (B) were ultrasonically degassed before use. The column was eluted under the following gradient conditions: 0–3 min, linear from 1% to 4% A; 3–5 min, linear from 4% to 30% A; 5–7 min 30% A; and 7.1–9 min, 1% A for equilibration of the column. The flow rate of the mobile phase was 0.30 mL/min, the column temperature was maintained at 25 °C, the sample-tray temperature was kept at 4 °C, and the injection volume was fixed at 2.0 µL.

### 3.4. MS Conditions

MS was performed on an Agilent 6460 Series Triple-Quadrupole Tandem Mass Spectrometer (Agilent Corporation, Santa Clara, CA, USA) equipped with an ESI source operated in positive-ion mode. The MS scanning mode ranged from *m*/*z* 50 to *m*/*z* 500. Quantitative analysis was conducted in SIM or MRM mode. The optimized source parameters were set as follows: drying gas temperature, 350 °C; drying gas flow, 10.0 L/min; nebulizing gas pressure, 35 psi; and capillary voltage, 4000 V.

### 3.5. Calibration Solutions

Standard stock solutions of all analytes, except for guanine, were prepared by dissolving each compound in an aqueous methanol solution (10%, *v*/*v*) at 0.5 mg/mL and stored at 4 °C. Guanine (0.1 mg/mL) was prepared in 0.1 mol/L HCl and then stored at 4 °C. The aforementioned standard solutions were prepared and diluted to appropriate concentrations with 10% (*v*/*v*) aqueous methanol for calibration-curve construction. Each calibration curve was generated by running samples at seven different concentrations in triplicate. In all reference solutions, the concentration of internal standard (2-chloroadenosine) was 1 µg/mL. Relative peak areas were plotted against the concentration of each analyte. Correlation coefficient was determined using a linear-regression model.

## 4. Conclusions

A rapid, sensitive, and reliable method for the quantitative analysis of nucleosides and nucleobases in *C. sinensis* was developed. The method was a combination of ultrasonic extraction in an ice bath and UHPLC–ESI–MS/MS analysis. Compared with a similar previously reported analytical method [7,10,25], our method was found to have greatly reduced analysis time, with all target analytes separated in 5.5 min, and relatively high sensitivity was achieved for most of the compounds. At the same time, the transformation of thermally unstable compounds during sample preparation was avoided. The method was successfully applied to the simultaneous determination of 13 nucleosides and nucleobases in 11 batches of *C. sinensis* from different regions in China. Analysis results showed that *C. sinensis* was rich in nucleoside compounds, and that the content of nucleoside compounds varied obviously in *C. sinensis* from different regions, thereby providing a theoretical basis for rational use of *C. sinensis* resources. In addition, the proposed method of UHPLC–ESI–MS/MS can be used to detect nucleosides in *C. sinensis* and to quantitatively analyze nucleosides or strong polar compounds in traditional Chinese medicines, as well as foods containing nucleosides or health care products.
